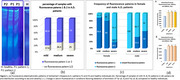# Specific Fluorescence Electrophoretic Patterns in the Serum of patients with Alzheimer's Disease. Correlation with Sex, Severity and amino acid fluorescence

**DOI:** 10.1002/alz70861_108938

**Published:** 2025-12-23

**Authors:** Dionysia Amanatidou, Vasileios Fouskas, Magda Tsolaki, Anna Anastasiou, Athanasia Papageorgiou, Phaedra Eleftheriou

**Affiliations:** ^1^ International Hellenic University, Thessaloniki Greece; ^2^ Aristotle University of Thessaloniki, Thessaloniki Greece

## Abstract

**Background:**

Fluorescence is an intrinsic property of proteins due to the presence of Trp, Tyr and Phe. Additionally, proteins bound to AGEs or proteins acting as carriers of fluorescing amino acids or other molecules may contribute to serum fluorescence.

In the present study, 60 serum samples of patients with mild(20), medium(20) and severe(20) Alzheimer’s Disease (A.D.) were electrophorized in non‐denatured conditions and the fluorescent patterns observed were compared with that of 20 samples of healthy individuals and other patients.

**Methods:**

Native PAGE was performed, and fluorescence was observed using a UV‐lamp. Serum fluorescence was also measured using an ELISA fluorimeter at conditions appropriate for the emission due to Trp, Tyr, Phe and AGEs.

**Results:**

Two specific fluorescence electrophoretic patterns(P1&P2) were observed in 75‐82% of A.D. patients of all severity groups. Among the fluorescence positive patients, P1 was observed in the majority of females with mild(88.8%) or medium (80%) disease while P2 was the dominating pattern in females with severe A.D.(72.7%). On the contrary, P2 was the dominate pattern in males with mild A.D.(100%), with its frequency to decrease in medium(66.7%) and severe (50%)disease. These patterns were not present in healthy individuals or diabetic or cancer patients tested but they were found in the serum of patients with other neurologic disorders some of which share common characteristics and comorbidity which A.D. such as suppression (pattern 1).

A statistically significant 30%‐38% increase was observed in total serum fluorescence under conditions favoring Trp(280/340nm) and Tyr(260/300nm) excitation/emission. The increase was also observed in both supernatant and precipitate following alkaline denaturation, with protein precipitate mostly being responsible for the observed fluorescence. Fluorescence at 340 nm (Trp) was the dominating one with much lower contribution of fluorescence at Tyr or AGEs. The appearance of specific fluorescent zones may indicate increase in concentration of a rich in Trp protein, change in protein conformation or/and in affinity to a fluorophore such as Trp.

**Conclusions:**

The results indicate that Alzheimer's Disease can be categorized in two groups differing in fluorescence electrophoretic pattern which can be correlated with differences in disease mechanism or comorbidities and could potentially be exploited in facilitating diagnosis and personalized treatment.